# The data of an all-solid-state acupuncture needle based potentiometric microelectrode for *in vivo* monitoring of calcium ions in rat cerebrospinal fluid

**DOI:** 10.1016/j.dib.2022.107949

**Published:** 2022-02-15

**Authors:** Jiali Zhai, Yaqun Zhang, Dongmei Zhao, Lijuan Kou, Guangtao Zhao

**Affiliations:** a School of Rehabilitation, Binzhou Medical University, Yantai 264003, PR China; b School of Basic Medicine, Binzhou Medical University, Yantai 264003, PR China; c School of Pharmacy, Binzhou Medical University, Yantai 264003, PR China

**Keywords:** Potentiometric microelectrodes, Acupuncture needle, Calcium, Spinal cord transection

## Abstract

These data contain the details of the fabrication of the calcium ion-selective microelectrode (Ca^2+^-ISμEs) modified with poly(3,4-ethylenedioxythiophene)-poly(sodium 4-styrenesulfonate) (PEDOT(PSS)) as solid contact. The electrochemical impedance spectroscopy was carried out for the investigation of the resistance of the Ca^2+^-ISμEs. The thickness of the solid contact and the calcium ion-selective membrane was investigated by SEM. Potential-time curve of the electrodeposition of the PEDOT/PSS film onto the surface of the microelectrodes under the applied current of 0.5 μA for 200 s was recorded. The proposed Ca^2+^-ISμE was optimized through conditioning in different CaCl_2_ solutions ranged from 1.0 × 10^−6^ to 3.1 × 10^−3^ M for different time before use. The anti-fouling property of the Ca^2+^-ISμEs against proteins was investigated through taking BSA as the model protein. The developed Ca^2+^-ISμEs were used for the *in vivo* monitoring of the calcium ions in rat cerebrospinal fluid under the stimuli of the spinal cord transection in a living animal.

## Graphical abstract

**Figure:**
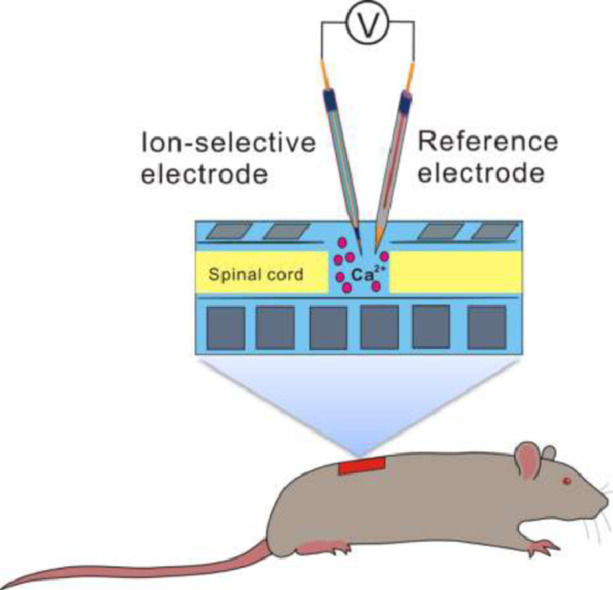

## Specifications Table

**Table:** 

Subject	Electrochemistry
Specific subject area	Electrochemical microsensors have been widely applied to solve the challenges in environmental chemistry, life science, and potentiometry based on polymeric membrane ion-selective electrodes has been widely used for selective and sensitive detection of cations and anions in biological and environmental samples. The developed all-solid-state potentiometric microelectrodes with the advantages of ease of preparation and robustness have a potential application in the brain research, marine science, and environmental chemistry.
Type of data	Figure
How the data were acquired	The thickness of the solid contact and the calcium ion-selective membrane covered onto the surface of the microelectrode was investigated through SEM, and the samples were sputtered with gold before measurement. The electrodeposition of the PEDOT(PSS), electrochemical impedance spectroscopy, and electromotive force (EMF) measurements were carried out at room temperature using an electrochemical workstation. The SEM images were obtained by an EVO LS15 (ZEISS, German). The modification of the PEDOT(PSS), electrochemical performance of the microelectrode, and the electromotive force (EMF) measurements were carried out though a CHI 660E electrochemical workstation (Shanghai Chenhua Apparatus Corporation, China).
Data format	Raw Analyzed
Description of data collection	The preparation of the microelectrode and the electrochemical measurements were all carried out at room temperature in a faraday cage, and the time interval of the signal recording was 0.1 s, and all the data points were collected for analysis.
Data source location	*Institution: Binzhou Medical University* *City/Town/Region: Yantai Laishan District* *Country: China* *Latitude and longitude (and GPS coordinates, if possible) for collected samples/data: 37˚ 27’ 33’’ N, 121˚ 27’ 28’’ E*
Data accessibility	Repository name: Mendeley Data Data identification number: 10.17632/nvk2frffm7.1 Direct URL to data: https://data.mendeley.com/datasets/nvk2frffm7/draft?a=29c7850f-94df-41be-8a09-351f2270e9d7
Related research article	J. L. Zhai, Y. Q. Zhang, D. M. Zhao, L. J. Kou, G. T. Zhao, *In vivo* monitoring of calcium ions in rat cerebrospinal fluid using an all-solid-state acupuncture needle based potentiometric microelectrode, Anal. Chim. Acta, 1191 (2022) 339209: 1-6. https://doi.org/10.1016/j.aca.2021.339209.

## Value of the Data

• These data contain the details of the fabrication of an all-solid-state Ca^2+^-ISμE for *in vivo* monitoring of calcium ions in CSF in a living animal, including the electrodeposition of the PEDOT(PSS) onto the surface of the microelectrode as conducting film, the resistance, and the potentiometric performance of the Ca^2+^-ISμE. These data provide a versatile method for the investigations of physiological and pathological of the central nervous system.
• The researchers who work on the *in vivo* monitoring of cations and anions in biology or microenvironment through ion-selective microelectrodes could benefit from these data.
• These data can provide help for further research of calcium change in other stage of the stress response of the neuron. Moreover, these data can also be used for other ions monitoring in a living animal through changing ion-selective membrane.
• The equation provided could be used for the resistance calculation of other type of electrodes and microelectrodes*.*

## 1 Data Description

**Fig. 1:**
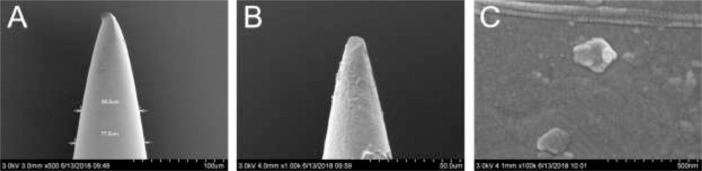
SEM images of the (A) bare acupuncture needle microelectrodes, (B) the microelectrodes modified with PEDOT(PSS), and (C) the magnification of PEDOT(PSS) film.

The figures named as “Fig. 1A”, “Fig. 1B”, and “Fig. 1C” refer to the SEM images of the (A) bare acupuncture needle microelectrodes, (B) the microelectrodes modified with PEDOT(PSS), and (C) the magnification of PEDOT(PSS) film (Fig. 1), and the Fig. 1A–C are from the research article [1].

**Fig. 2:**
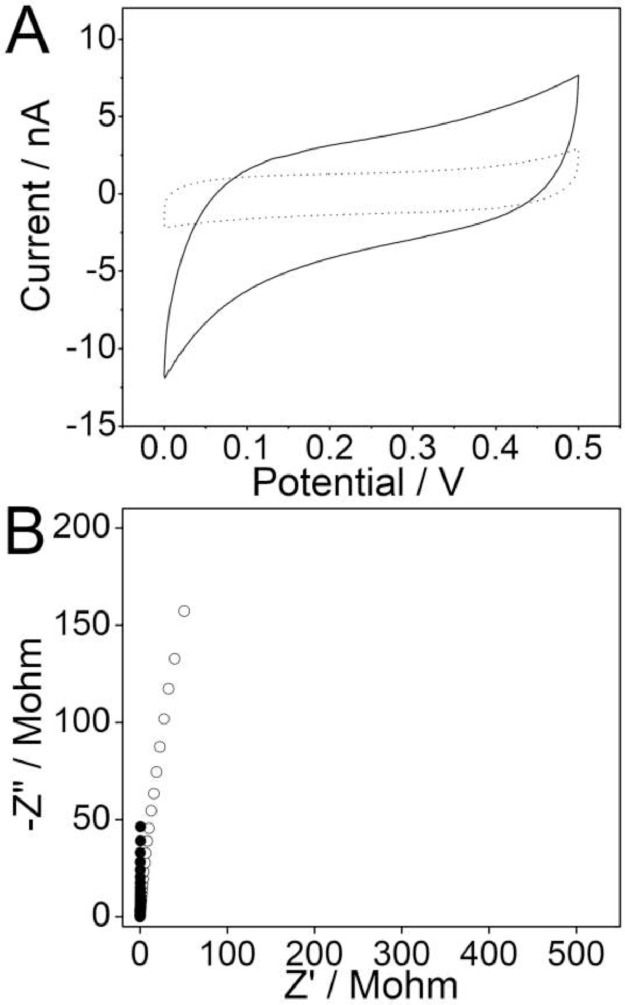
(A) Cyclic voltammograms recorded in 0.1 M KCl for the bare acupuncture needle microelectrodes (solid line) and the acupuncture needle microelectrodes modified with PEDOT(PSS) composite (short dot). Scan rate, 50 mV/s. (B) Impedance spectra for the bare acupuncture needle microelectrodes (○) and the microelectrodes modified with PEDOT(PSS) (●) in 0.1 M KCl solution at the open-circuit potential. Scan rate, 50 mV/s, frequency range, 0.01 Hz–10 kHz, excitation amplitude, 100 mV.

The data files named as “Figure-2-A-short dot-raw data” and “Figure-2-A-solid line-raw data” refer to the raw data of the cyclic voltammograms recorded in 0.1 M KCl for the bare acupuncture needle microelectrodes (solid line) and the acupuncture needle microelectrodes modified with PEDOT(PSS) composite (short dot). Scan rate, 50 mV/s (Fig. 2A), and the Fig. 2A is from the research article [1].

The data files named as “Figure-2-B-circle-raw data” and “Figure-2-B-dot-raw data” refer to the raw data of the impedance spectra for the bare acupuncture needle microelectrodes (○) and the microelectrodes modified with PEDOT(PSS) (●) in 0.1 M KCl solution at the open-circuit potential. Frequency range, 0.01 Hz–10 kHz, excitation amplitude, 100 mV (Fig. 2B), and the Fig. 2B is from the research article [1].

**Fig. 3:**
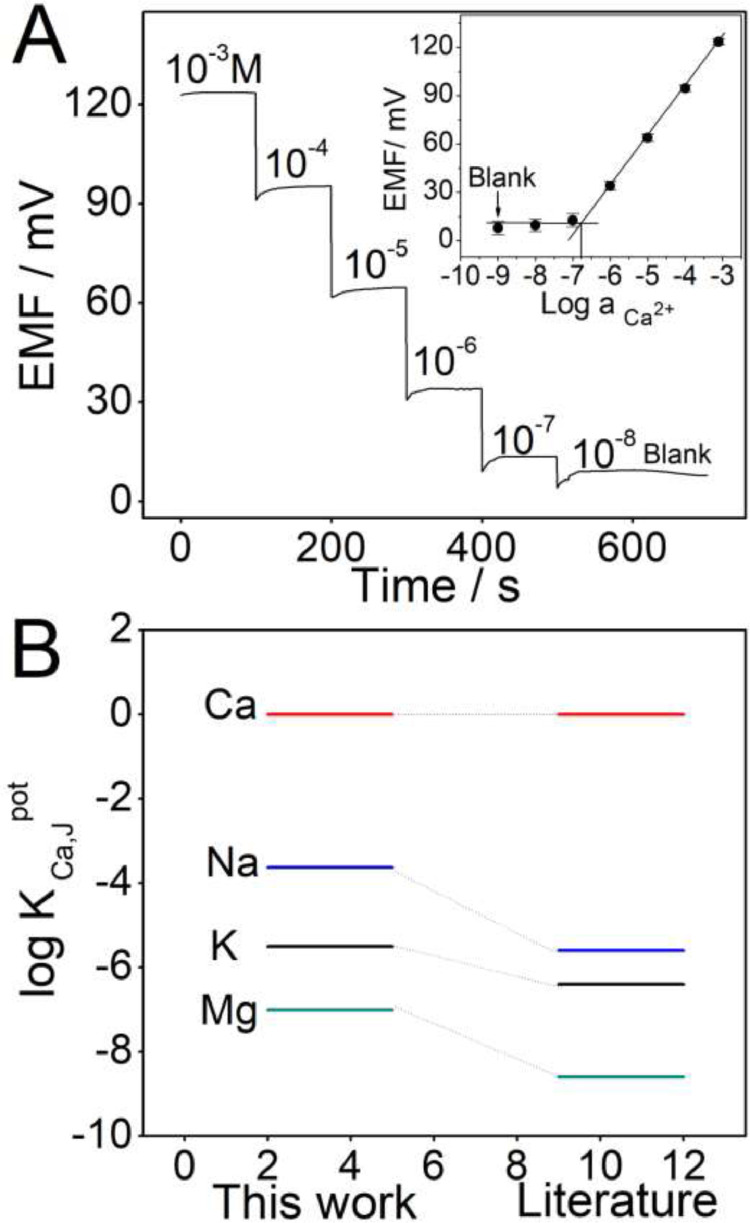
(A) Potential time trace of the Ca^2+^-ISμE in CaCl_2_ solutions range from 1.0 × 10^−8^ to 3.1 × 10^−3^ M; the inset was the calibration curve of the Ca^2+^-ISμE in CaCl_2_ solutions. (B) Comparison of the selectivity coefficients for the Ca^2+^-ISμE with the separate solution method [28] and the literature [29].

The data file named as “Figure-3-A-raw data” refers to the raw data of the potential time trace of the Ca^2+^-ISμE in CaCl_2_ solutions range from 1.0 × 10^−8^ to 3.1 × 10^−3^ M (Fig. 3A), and the data file named as “Figure-3-A-analyzed data” refer to the analyzed data of the inset of the Fig. 3A, which was the calibration curve of the Ca^2+^-ISμE in CaCl_2_ solutions. The data file named as “Figure-3-B-selectivity coefficient analyzed data” refers to the analyzed data of the comparison of the selectivity coefficients for the Ca^2+^-ISμE with the separate solution method and the literature (Fig. 3B), and the Fig. 3 is from the research article [1].

**Fig. 4:**
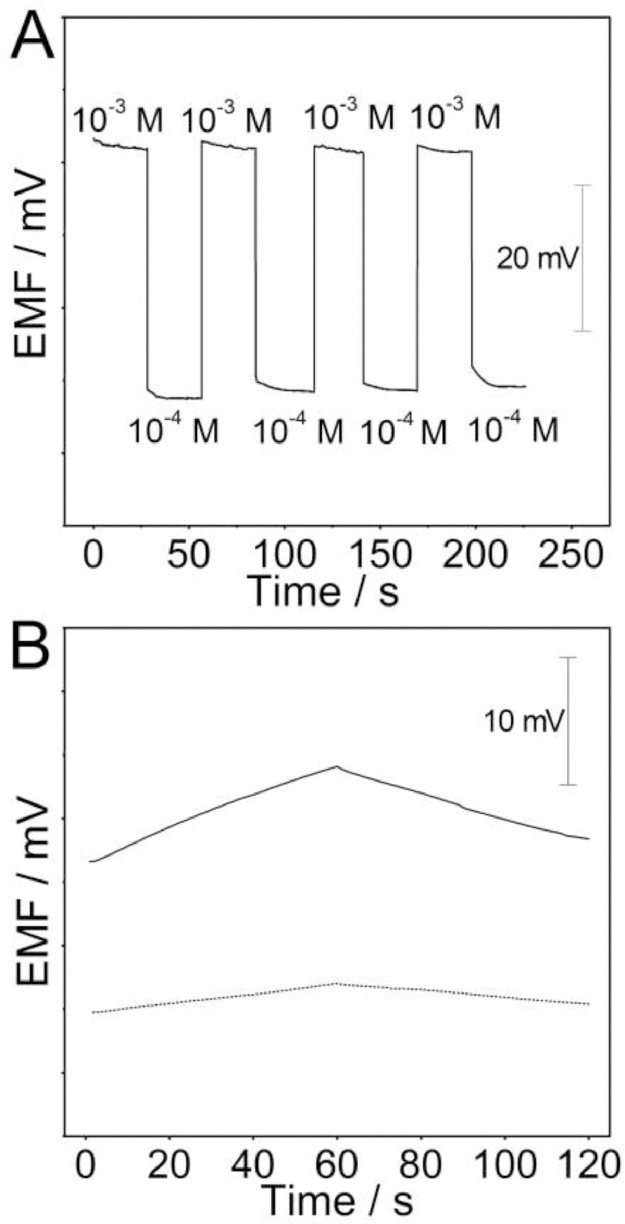
(A) Potential reproducibility of the Ca^2+^-ISμE evaluated by alternatively measuring 10^−4^ and 10^−3^ M CaCl_2_ (*n* = 4). (B) Chronopotentiograms for the bare acupuncture needle microelectrodes (line) and the microelectrodes modified with PEDOT(PSS) (dot) film recorded in 1.0 × 10^−5^ M CaCl_2_. The applied current was +0.01 nA for 60 s and −0.01 nA for another 60 s.

The data file named as “Figure-4-A-raw data” refers to the raw data of the potential reproducibility of the Ca^2+^-ISμE evaluated by alternatively measuring 10^−4^ and 10^−3^ M CaCl_2_ (*n* = 4) (Fig. 4A). The data files named as “Figure-4-B-dot-raw data” and “Figure-4-B-solid line raw data” refer to the raw data of chronopotentiograms for the bare acupuncture needle microelectrodes (line) and the microelectrodes modified with PEDOT(PSS) (dot) film recorded in 1.0 × 10^−5^ M CaCl_2_. The applied current was +0.01 nA for 60 s and −0.01 nA for another 60 s (Fig. 4B), and the Fig. 4 is from the research article [1].

**Fig. 5:**
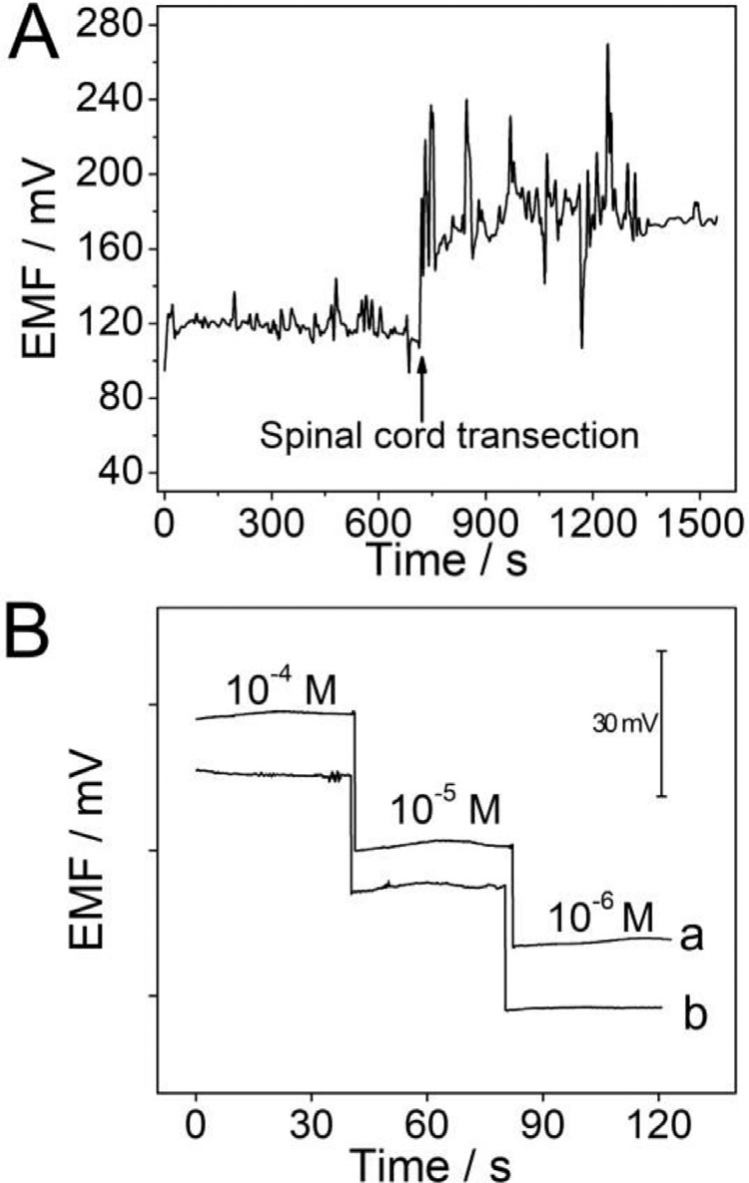
(A) Potential responses of the Ca^2+^-ISμE toward calcium change before and after spinal cord transection. (B) Potential responses of the Ca^2+^-ISμE in CaCl_2_ solutions range from 10^−6^ to 10^−4^ M before (a) and after (b) the electrode was implanted in CSF for *in vivo* measurement.

The data file named as “Figure-5A-raw data” refers to the raw data of potential responses of the Ca^2+^-ISμE toward calcium change before and after spinal cord transection (Fig. 5A). The data file named as “Figure-5B-a-raw data” and “Figure-5B-b-raw data” refer to the raw data of potential responses of the Ca^2+^-ISμE in CaCl_2_ solutions range from 1 × 10^−6^ to 1 × 10^−4^ M before (a) and after (b) the electrode was implanted in CSF for *in vivo* measurement (Fig. 5B), and the Fig. 5 is from the research article [1].

The data file named as “Figure-S1-raw data” refers to the raw data of Fig. S1, which is the potential-time curve of the electrodeposition of the PEDOT/PSS film onto the surface of the microelectrodes under the applied current of 0.5 μA for 200 s, and the Fig. S1 is from the supporting information of the research article [1].

The figure named as “Figure-S2” refers to the SEM images of the full length of the tip of the microelectrode modified with PEDOT/PSS film exposed to the glass capillary (A), the PEDOT(PSS) film (B), and the Ca^2+^-ISM covered onto the surface of the PEDOT(PSS) film (C), and the Fig. S2 is from the supporting information of the research article [1].

The data file named as “Figure-S3-analyzed data” refers to the analyzed data of Fig. S3, which is the calibration curve of the Ca^2+^-ISμEs in CaCl_2_ solutions per decade after conditioned in CaCl_2_ solutions in the activity range from 1.0 × 10^−6^ to 3.1 × 10^−3^ M for 0.5 h, 1 h, and 2 h, respectively, and the Fig. S3 is from the supporting information of the research article [1].

The data file named as “Figure-S4 analyzed data” refers to the analyzed data of Fig. S4, which is the calibration curve of the Ca^2+^-ISμEs in CaCl_2_ solutions per decade after conditioned in CaCl_2_ solutions in the activity range from 1.0 × 10^−6^ to 3.1 × 10^−3^ M for 0.5, 1, and 2 h, respectively, and the Fig. S4 is from the supporting information of the research article [1].

The data file named as “Figure-S5-raw data” refers to the raw data of Fig. S5, which is the impedance spectra for the Ca^2+^-ISμE modified with PEDOT(PSS) in 0.1 M KCl solution at the open-circuit potential. Frequency range, 0.01 Hz–10 kHz, excitation amplitude, 100 mV, and the Fig. S5 is from the supporting information of the research article [1].

The data file named as “Figure-S6-raw data” refers to the raw data of Fig. S6, which is the potential time trace of the Ca^2+^-ISμE towards Ca^2+^ in an artificial cerebral spinal fluid solution per decade and the successive addition of BSA (first 8 additions, each addition, 2.5 mg/mL; second 7 additions, each addition, 10 mg/mL), and the Fig. S6 is from the supporting information of the research article [1].

The data file named as “Figure-S7-raw data” refers to the raw data of Fig. S7, which is the potential time trace of the Ca^2+^-ISμE in CaCl_2_ solutions range from 1.0 × 10^−5^ to 3.1 × 10^−3^ M after the electrodes immersed in 40 mg/mL BSA in the background of artificial cerebral spinal fluid without calcium for 3 h, and the Fig. S7 is from the supporting information of the research article [1].

## 2 Experimental Design, Materials and Methods

Acupuncture needle is a common tool in traditional Chinese medicine, and it could be an ideal material for the development of all-solid-state microelectrode for its small tip with micro size. Taken the diameter, length, material, and electrical conductivity into considering, we chose the acupuncture needle made of stainless steel with silver plating with a tip diameter of less than 80 μm (Suzhou Medical Appliance Factory, China) at last. An acupuncture needle was carefully inserted into a pre-drawn glass capillary tube, and the tip of the acupuncture needle exposed out of the glass capillary was about 50–100 μm, which can be controlled by the naked eye. The end of the acupuncture needle and its tip were both fixed by casting non-conducting epoxy glue at the stem end and the tip of the capillary tube. After dried in the air, the prepared acupuncture needle microelectrodes were immersed in 1.0 M HNO_3_ for 15 min, and then cleaned ultrasonically in deionized water and ethanol for 5 min, respectively [1].

The modification of the solid contact onto the surface of the microelectrode could decrease the high resistance of the microelectrode, and also could increase the double layer capacitance. We chose galvanostatic electrochemical polymerization because of the advantages of simple operation and time saving. The electrodeposition system consisted of the prepared microelectrode as the working electrode, an Ag/AgCl/3 M KCl microelectrode as the reference electrode, and a Pt wire as the counter electrode. The polymerization was carried out in a deaerated solution containing 0.1 M EDOT and 0.01 M NaPSS. A constant current of 0.5 μA was applied for 200 s to produce polymerization charges of 100 μC [2], [3]. The microelectrodes were left to dry at room temperature for at least 24 h after electrodeposition.

100 mg of the membrane components, including 1.3 wt% Ca^2+^ ionophore Ⅳ (ETH5234), 0.6 wt% NaTFPB, 65.3 wt% *o*-NPOE, and 32.8 wt% PVC were dissolved in 0.8 mL of THF. The calcium ion-selective membrane was very difficult to be applicated onto the surface of the microelectrode due to its small tip. Therefore, the tip of the proposed microelectrode was dipped into the Ca^2+^ ion-selective membrane cocktail solution for six times, and the microelectrode was left in the air to dry for 15 min at room temperature after each dip, and the dipping times has also been optimized (data not shown). After being dried, the prepared microelectrodes denoted as Ca^2+^-ISμEs were conditioned in CaCl_2_ solution before use. The thickness of the PEDOT(PSS) film and the Ca^2+^ ion-selective membrane was also investigated.

The solution of CaCl_2_ and time used for conditioning of the Ca^2+^-ISμEs before use was also optimized. For comparison, the Ca^2+^-ISμE was prepared by coating the ion-selective membrane on the bare acupuncture needle microelectrode.

Electromotive force (EMF) measurements were carried out at room temperature using a CHI 660E electrochemical workstation (Shanghai Chenhua Apparatus Corporation, China). An Ag/AgCl/3 M KCl microelectrode with a diameter of 10 μm was used as the reference microelectrode [4]. Before measurement, the long-term potential stability of the Ca^2+^-ISμEs also need to be investigated. The existence of the PEDOT(PSS) could avoide the water layer between the calcium ion-selective membrane and the surface of the microelectrode. Moreover, the total resistance and low-frequency capacitance of the Ca^2+^-ISμEs could also be calculated. The selectivity coefficients of the Ca^2+^-ISμEs toward various interfering ions in CSF need to be examined through the separate solution method, such as K^+^, Na^+^, and Mg^2+^ [5], [6].

In order to investigate the resistance of the microelectrodes before and after the modification of the PEDOT(PSS), electrochemical impedance spectroscopy (EIS) was carried out in 0.1 M KCl solution by using a three-electrode system, composing of the proposed microelectrode as working electrode, an Ag/AgCl/3 M KCl microelectrode as reference electrode and a Pt wire as counter electrode.

The anti-fouling property of the prepared Ca^2+^-ISμEs need to be investigated before applied for *in vivo* monitoring of calcium ions, as there would be many kinds of proteins in CSF.

Adult Sprague-Dawley rats were chose as animal model for the *in vivo* measurement of calcium ions under stimuli. Adult Sprague-Dawley rats were purchased from the Pengyue Experimental Animal Breeding Co., Ltd, Jinan, China (License number SCXK (lu) 2019003). The rats were housed under a standard 12 h light/dark cycle, and fed with sterile water and standard laboratory chow. The EMF measurements were carried out to check whether the Ca^2+^-ISμEs could also maintain Nernstian response after *in vivo* measurements.

## Ethics Statements

The experiments were complied with the ARRIVE guidelines and were carried out in accordance with the U.K. Animals (Scientific Procedures) Act, 1986 and associated guidelines.

The rats are all male.

## CRediT authorship contribution statement

**Jiali Zhai:** Data curation, Writing – original draft. **Yaqun Zhang:** Data curation. **Dongmei Zhao:** Formal analysis. **Lijuan Kou:** Validation. **Guangtao Zhao:** Writing – review & editing.

## Declaration of Competing Interest

The authors declare that they have no known competing financial interests or personal relationships that could have appeared to influence the work reported in this paper. The authors declare the following financial interests/personal relationships which may be considered as potential competing interests.

## Appendix Supplementary materials

Image, application 1
