# Supplementary material for: The data of an all-solid-state acupuncture needle based potentiometric microelectrode for in vivo monitoring of calcium ions in rat cerebrospinal fluid
Source: Data Brief. 2022 Feb 15;41:107949. doi: 10.1016/j.dib.2022.107949 (PMC8866674; doi:10.1016/j.dib.2022.107949)
Supplement: Supplementary file 1 [file mmc1.docx]

*In vivo* monitoring of calcium ions in rat cerebrospinal fluid using an all-solid-state [acupuncture](javascript:;) [needle](javascript:;) based potentiometric microelectrode

Jiali Zhai^a^, Yaqun Zhang^b^, Dongmei Zhao^b^, Lijuan Kou^c^, and Guangtao Zhao^b*^

*^a^ School of Rehabilitation, Binzhou Medical University, Yantai, P.R. 264003, China;*

*^b^ School of Basic Medicine, Binzhou Medical University, Yantai, P.R. 264003, China.*

*^c^ School of Pharmacy, Binzhou Medical University, Yantai, P.R. 264003, China.*

** Corresponding author. Tel.: +86 535 6913213; Fax: +86 535 6913246.*

*E-mail address: gtzhao@bzmc.edu.cn (G. Zhao)*

As shown in Fig. S3, the Ca^2+^-ISμE was conditioned in the CaCl_2_ solutions ranged from 1.0 × 10^-6^ to 3.1 × 10^-3^ M for different time. The Ca^2+^-ISμE could reach Nernstian response after conditioned in 1 × 10^-4^ M and 3.1 × 10^-3^ M CaCl_2_ solutions for 1 h, while it would take longer time for the Ca^2+^-ISμE conditioned in 1.0 × 10^-5^ M and 1.0 × 10^-6^ M CaCl_2_ solutions to reach Nernstian response. As the influence of the CaCl_2_ solutions of different concentrations on the detection limit of the Ca^2+^-ISμE was not found, and take time consuming into account, the Ca^2+^-ISμE was conditioned in 1 × 10^-4^ M CaCl_2_ solution for 1 h before use.


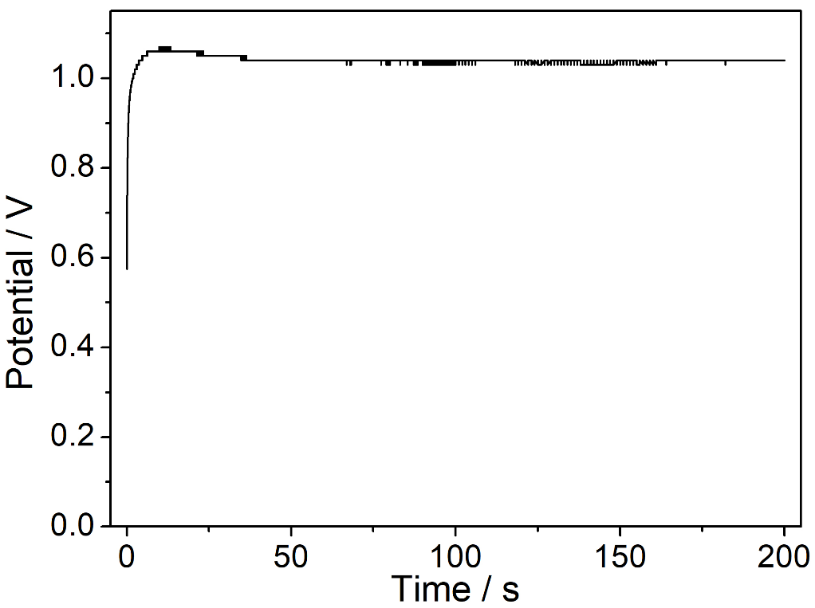


Fig. S1. Potential-time curve of the electrodeposition of the PEDOT/PSS film onto the surface of the microelectrodes under the applied current of 0.5 μA for 200 s.


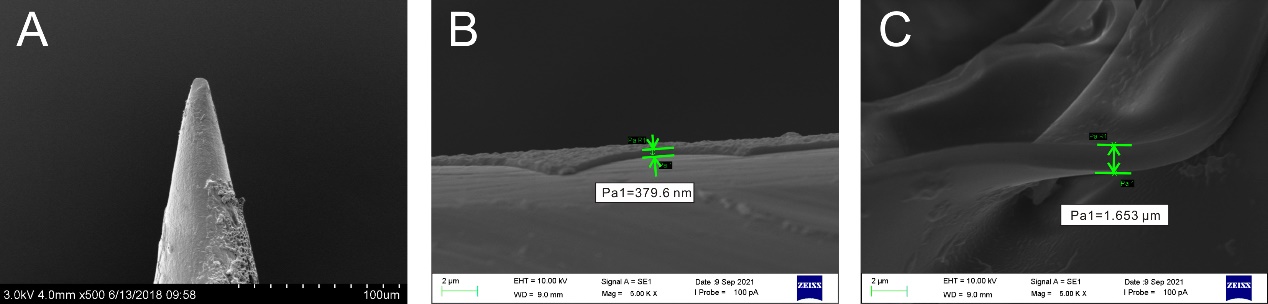


Fig. S2. SEM images of the full length of the tip of the microelectrode modified with PEDOT/PSS film exposed to the glass capillary (A), the AuμE/PEDOT(PSS) film (B), and the Ca^2+^-ISM covered onto the surface of the PEDOT(PSS) film (C).


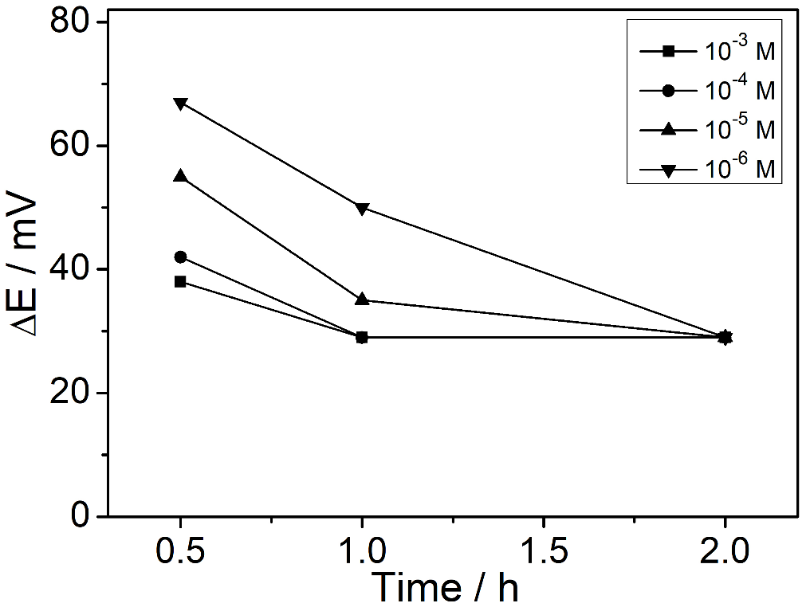


Fig. S3. The calibration curve of the Ca^2+^-ISμEs in CaCl_2_ solutions per decade after conditioned in CaCl_2_ solutions in the activity range from 1.0 × 10^-6^ to 3.1 × 10^-3^ M for 0.5, 1h, and 2 h, respectively.


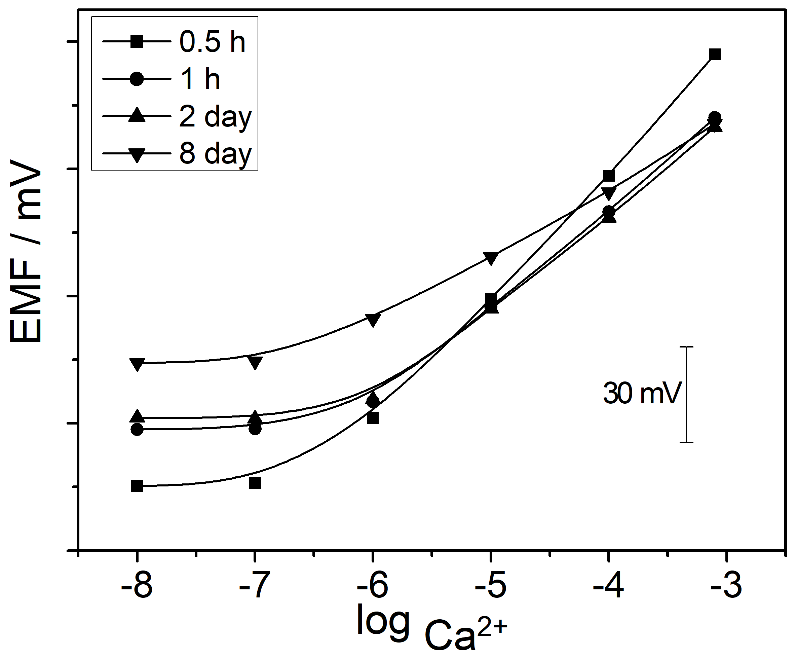


Fig. S4. Calibration curves of the Ca^2+^-ISμE in CaCl_2_ solutions in the activity range of 1.0 × 10^-8^ - 3.1× 10^-3^ M.


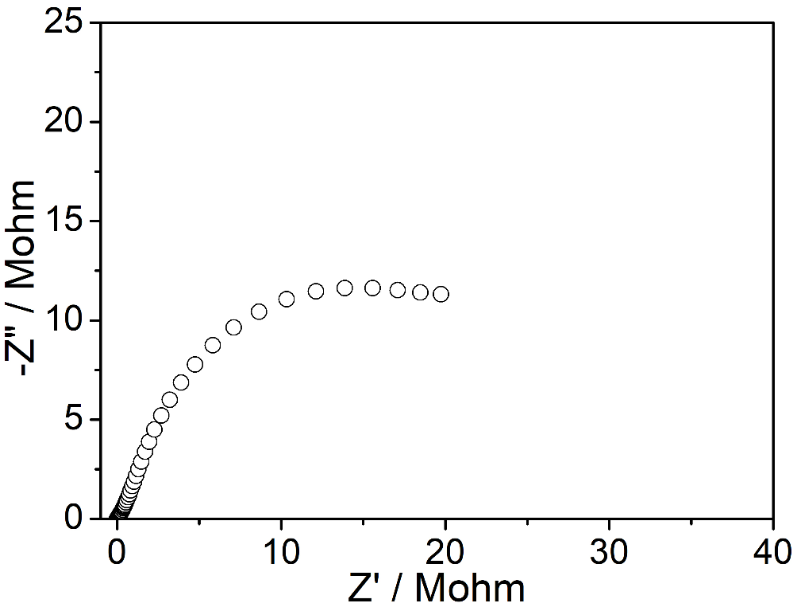


Fig. S5. Impedance spectra for the Ca^2+^-ISμE modified with PEDOT(PSS) in 0.1 M KCl solution at the open-circuit potential. Frequency range, 0.01 Hz to 10 kHz, excitation amplitude, 100 mV.


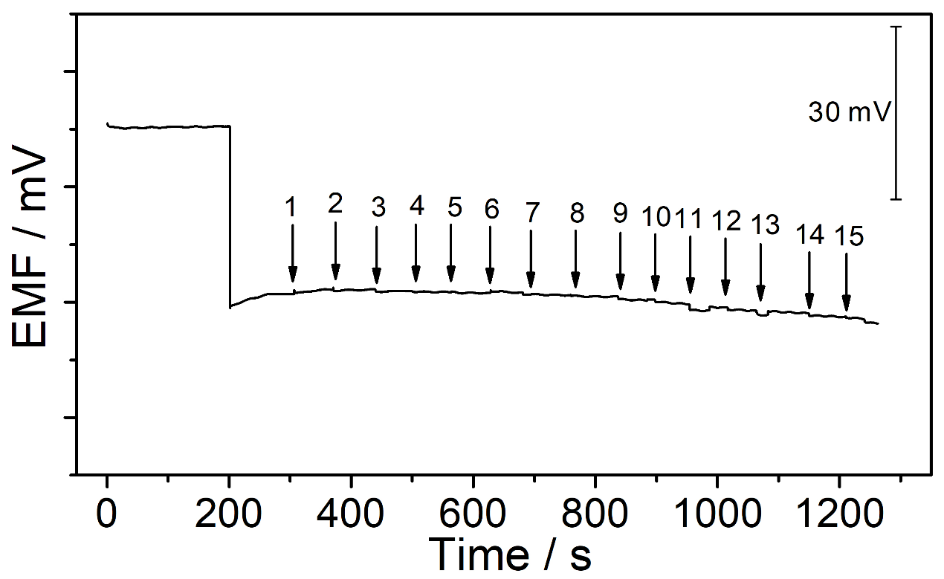


Fig. S6. Potential time trace of the Ca^2+^-ISμE towards Ca^2+^ in an artificial cerebral spinal fluid solution per decade and the successive addition of BSA (first 8 additions, each addition, 2.5 mg/mL; second 7 additions, each addition, 10 mg/mL)


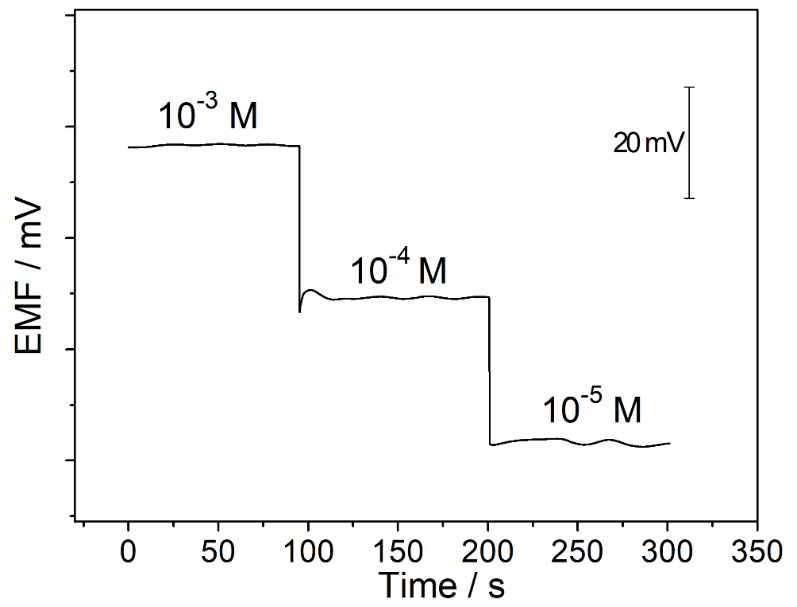


Fig. S7. Potential time trace of the Ca^2+^-ISμE in CaCl_2_ solutions range from 1.0 × 10^-5^ to 3.1 × 10^-3^ M after the electrodes immersed in 40 mg/mL BSA in the background of artificial cerebral spinal fluid without calcium for 3 hours.
